# Tris(2,2′-bipyridine-κ^2^ *N*,*N*′)cobalt(II) bis­(hexa­fluoridophosphate)

**DOI:** 10.1107/S1600536812050234

**Published:** 2012-12-15

**Authors:** Ayfer Menteş, Kuldip Singh

**Affiliations:** a Aksaray University, Faculty of Arts and Sciences, Department of Chemistry, 68100 Aksaray, Turkey; b Department of Chemistry, Leicester University, Leicester LE1 7RH, England

## Abstract

In the title compound, [Co(C_10_H_8_N_2_)_3_](PF_6_)_2_, the Co^II^ atom is coordinated by the six N atoms of three 2,2′-bipyridyl ligands and adopts a highly distorted octa­hedral geometry. The crystal used was a merohedral twin, the refined ratio of twin components being 0.820 (1):0.180 (1). The crystal structure features weak C—H⋯F inter­actions, forming a three-dimensional network.

## Related literature

For related structures, see: Chygorin *et al.* (2012 ▶); Liu *et al.* (2008 ▶, 2010 ▶).
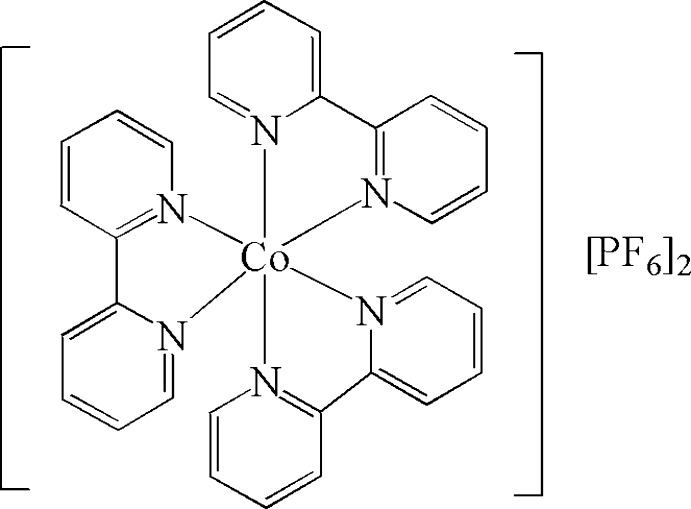


## Experimental

### 

#### Crystal data

[Co(C_10_H_8_N_2_)_3_](PF_6_)_2_
*M* *_r_* = 817.42
Trigonal, 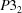

*a* = 10.3524 (18) Å
*c* = 26.140 (6) Å
*V* = 2426.2 (8) Å^3^
*Z* = 3
Mo *K*α radiation
μ = 0.73 mm^−1^
*T* = 150 K
0.35 × 0.16 × 0.13 mm

#### Data collection

Bruker APEX 2000 CCD area-detector diffractometer
Absorption correction: multi-scan (*SADABS*; Bruker, 2005 ▶) *T*_min_ = 0.581, *T*_max_ = 0.862
19252 measured reflections
6285 independent reflections
5344 reflections with *I* > 2σ(*I*)
*R*_int_ = 0.087

#### Refinement

*R*[*F* ^2^ > 2σ(*F* ^2^)] = 0.049
*wR*(*F* ^2^) = 0.088
*S* = 0.94
6285 reflections
461 parameters
1 restraint
H-atom parameters constrained
Δρ_max_ = 0.30 e Å^−3^
Δρ_min_ = −0.31 e Å^−3^
Absolute structure: Flack (1983 ▶), 3114 Friedel pairs
Flack parameter: 0.010 (18)

### 

Data collection: *APEX2* (Bruker, 2005 ▶); cell refinement: *SAINT* (Bruker, 2005 ▶); data reduction: *SAINT*; program(s) used to solve structure: *SHELXS97* (Sheldrick, 2008 ▶); program(s) used to refine structure: *SHELXL97* (Sheldrick, 2008 ▶); molecular graphics: *SHELXTL* (Sheldrick, 2008 ▶); software used to prepare material for publication: *SHELXTL*.

## Supplementary Material

Crystal structure: contains datablock(s) I, global. DOI: 10.1107/S1600536812050234/lr2090sup1.cif

Structure factors: contains datablock(s) I. DOI: 10.1107/S1600536812050234/lr2090Isup2.hkl

Additional supplementary materials:  crystallographic information; 3D view; checkCIF report

## Figures and Tables

**Table 1:** Hydrogen-bond geometry (Å, °)

*D*—H⋯*A*	*D*—H	H⋯*A*	*D*⋯*A*	*D*—H⋯*A*
C24—H24⋯F2^i^	0.95	2.49	3.324 (7)	146
C23—H23⋯F4^i^	0.95	2.55	3.253 (7)	131
C18—H18⋯F11^ii^	0.95	2.52	3.149 (6)	124
C13—H13⋯F10^iii^	0.95	2.50	3.208 (6)	131
C10—H10⋯F11^iv^	0.95	2.51	3.265 (6)	137
C9—H9⋯F7^iv^	0.95	2.33	3.136 (6)	142
C7—H7⋯F8^v^	0.95	2.38	3.160 (7)	139
C2—H2⋯F2^vi^	0.95	2.33	3.081 (7)	136

Symmetry codes: (i) 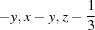; (ii) 
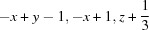
; (iii) 
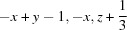
; (iv) 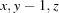; (v) 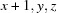; (vi) 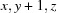.

**Fig. 1.**
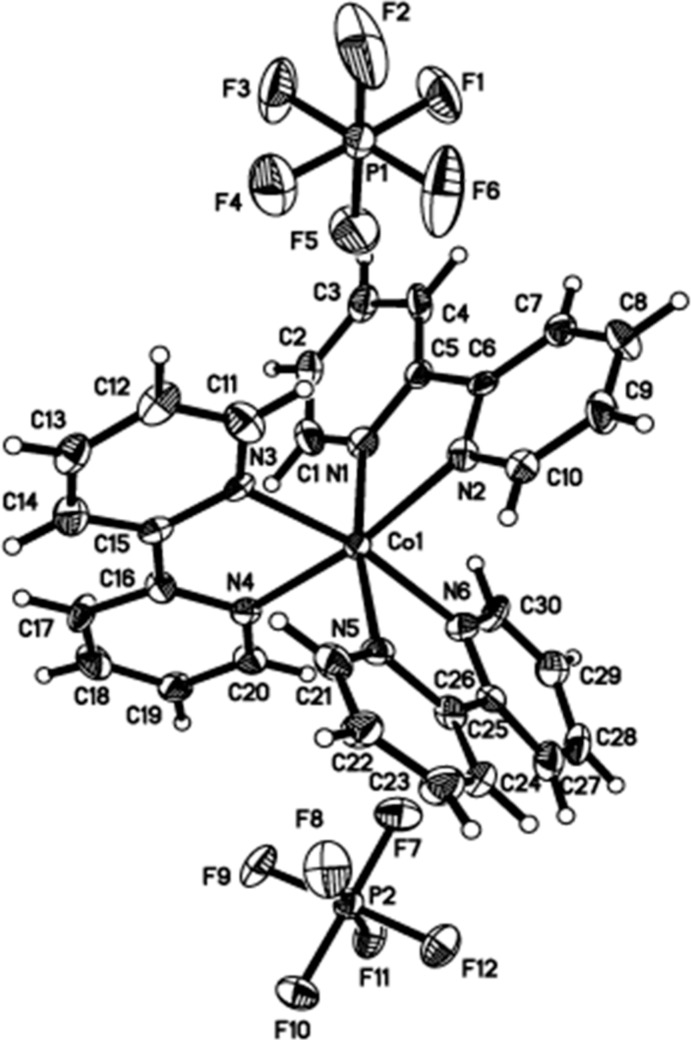
The molecular structure of (1). Displacement ellipsoids are drawn at the 30% probability level.

**Fig. 2.**
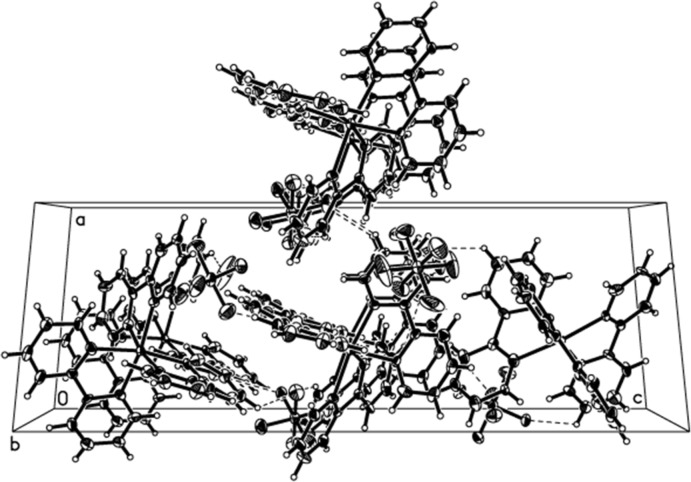
A view of the crystal packing of the title compound.

## supplementary crystallographic information

### Comment

The reaction of Cobalt(II) pentafluoropropionate with NaPF_6_ in the presence of 2,2'-bipyridine yields the coordination compound *tris*(bipyridine)cobalt(II) hexafluorophosphate (1), [Co(bipy)_3_][PF_6_]_2_. Crystals were grown by allowing slow evaporation of the complex in ethanol. The title complex crystallizes in the space group P3(2) in contrast to the related compound [Co(bipy)_3_][Mo_6_O_19_]_2_ which crystallizes in *P*2_1_/*n* (Liu *et al.*, 2010). The structure of (1) is shown in Fig. 1. Within the divalent complex cations, the cobalt atoms are each surrounded by six N atoms of three chelating bipy ligands to complete a distorted octahedral coordination with d(Co—N) = 2.098 (7) – 2.149 (8) Å, the *cis* and *trans* N—Co—N bond angles in the range 76.6 (3) – 96.9 (3) and 167.6 (3) – 170.5 (3)°, respectively. Such distances are similar to those found in other related structures (Liu *et al.*, 2008, Chygorin *et al.* 2012). There has been great interest in homoleptic imine complexes, because of great potential applications in many fields such as, catalysis, material science and medicine. The crystal packing of the title compound is presented in Fig. 2. The crystal structure is stabilized by weak intermolecular C—H···F bonds.

### Experimental

*Tris*(bipyridine)cobalt(II) hexafluorophosphate was prepared by stirring a mixture of a solution containing Co(O_2_CC_2_F_5_)_2_ (0.100 g, 0.25 mmol) and bipyridine (0.120 g, 0.77 mmol) in ethanol (10 ml). The reaction mixture was stirred for 2 h. After this time NaPF_6_ (0.100 g, 0.59 mmol) was added and stirred for 1 h. Solid of title compound was obtained by slow evaporation of an ethanol solution in refrigerator (Yield 0.150 g, 79%; m.p. 594 K). Yellow block crystals were obtained in acetonitrile/ethanol (1:1) solution after few days. ATR-IR: 1603 ν(C=N), 1566 ν(C=C) cm^-1^.

### Refinement

Hydrogen atoms were included in calculated positions (C—H = 0.95 Å) riding on the bonded atom with isotropic displacement parameters set to 1.2Ueq(C) for all hydrogen atoms. All non-H atoms were refined with anisotropic displacement ellipsoids. Merohedral twinning is indicated and applying the twin instruction [TWIN 0 1 0, 1 0 0, 0 0 - 1] with a BASF parameter in *SHELXTL* (Sheldrick, 2008), the R1 value drops to 0.049 (0.0886 without TWIN Instruction) and wR2 value drops to 0.0883 (0.2284 without TWIN instruction).

### Crystal data

**Table:**

[Co(C_10_H_8_N_2_)_3_](PF_6_)_2_	*D*_x_ = 1.678 Mg m^−^^3^
*M**_r_* = 817.42	Melting point: 594 K
Trigonal, *P*3_2_	Mo *K*α radiation, λ = 0.71073 Å
Hall symbol: P 32	Cell parameters from 812 reflections
*a* = 10.3524 (18) Å	θ = 2.3–28.2°
*c* = 26.140 (6) Å	µ = 0.73 mm^−^^1^
*V* = 2426.2 (8) Å^3^	*T* = 150 K
*Z* = 3	Needle, yellow
*F*(000) = 1233	0.35 × 0.16 × 0.13 mm

### Data collection

**Table:**

Bruker APEX 2000 CCD area-detector diffractometer	6285 independent reflections
Radiation source: fine-focus sealed tube	5344 reflections with *I* > 2σ(*I*)
Graphite monochromator	*R*_int_ = 0.087
phi and ω scans	θ_max_ = 26.0°, θ_min_ = 0.8°
Absorption correction: multi-scan (*SADABS*; Bruker, 2005)	*h* = −12→12
*T*_min_ = 0.581, *T*_max_ = 0.862	*k* = −12→12
19252 measured reflections	*l* = −32→32

### Refinement

**Table:**

Refinement on *F*^2^	Secondary atom site location: difference Fourier map
Least-squares matrix: full	Hydrogen site location: inferred from neighbouring sites
*R*[*F*^2^ > 2σ(*F*^2^)] = 0.049	H-atom parameters constrained
*wR*(*F*^2^) = 0.088	*w* = 1/[σ^2^(*F*_o_^2^) + (0.0279*P*)^2^] where *P* = (*F*_o_^2^ + 2*F*_c_^2^)/3
*S* = 0.94	(Δ/σ)_max_ = 0.002
6285 reflections	Δρ_max_ = 0.30 e Å^−^^3^
461 parameters	Δρ_min_ = −0.31 e Å^−^^3^
1 restraint	Absolute structure: Flack (1983), 3114 Friedel pairs
Primary atom site location: structure-invariant direct methods	Flack parameter: 0.010 (18)

### Special details

**Table:**

Experimental. SADABS (Bruker, 2005). Absorption correction based on 7715 reflections;Rint 0.1489 before correction and 0.0570 after.
Geometry. All e.s.d.'s (except the e.s.d. in the dihedral angle between two l.s. planes) are estimated using the full covariance matrix. The cell e.s.d.'s are taken into account individually in the estimation of e.s.d.'s in distances, angles and torsion angles; correlations between e.s.d.'s in cell parameters are only used when they are defined by crystal symmetry. An approximate (isotropic) treatment of cell e.s.d.'s is used for estimating e.s.d.'s involving l.s. planes.
Refinement. Refinement of *F*^2^ against ALL reflections. The weighted *R*-factor *wR* and goodness of fit *S* are based on *F*^2^, conventional *R*-factors *R* are based on *F*, with *F* set to zero for negative *F*^2^. The threshold expression of *F*^2^ > σ(*F*^2^) is used only for calculating *R*-factors(gt) *etc*. and is not relevant to the choice of reflections for refinement. *R*-factors based on *F*^2^ are statistically about twice as large as those based on *F*, and *R*- factors based on ALL data will be even larger.

### Fractional atomic coordinates and isotropic or equivalent isotropic displacement parameters (Å^2^)

**Table:**

	*x*	*y*	*z*	*U*_iso_*/*U*_eq_	
Co1	0.31501 (7)	0.63587 (6)	0.16543 (3)	0.02682 (14)	
N1	0.5410 (4)	0.7831 (4)	0.18887 (15)	0.0288 (9)	
N2	0.4286 (4)	0.5271 (4)	0.13828 (15)	0.0286 (9)	
N3	0.2500 (4)	0.5252 (4)	0.23714 (14)	0.0278 (9)	
N4	0.2286 (4)	0.7570 (4)	0.20398 (14)	0.0270 (9)	
N5	0.1048 (4)	0.4858 (4)	0.13110 (14)	0.0285 (9)	
N6	0.3326 (4)	0.7329 (4)	0.09234 (14)	0.0321 (9)	
C1	0.5893 (5)	0.9086 (5)	0.21651 (19)	0.0322 (11)	
H1	0.5220	0.9440	0.2229	0.039*	
C2	0.7309 (5)	0.9875 (6)	0.23577 (18)	0.0339 (12)	
H2	0.7617	1.0756	0.2552	0.041*	
C3	0.8278 (6)	0.9347 (6)	0.22598 (19)	0.0426 (13)	
H3	0.9265	0.9853	0.2392	0.051*	
C4	0.7788 (6)	0.8074 (6)	0.19675 (19)	0.0394 (13)	
H4	0.8443	0.7703	0.1895	0.047*	
C5	0.6371 (5)	0.7354 (5)	0.17835 (17)	0.0270 (10)	
C6	0.5765 (5)	0.5992 (5)	0.14565 (17)	0.0271 (10)	
C7	0.6663 (6)	0.5535 (6)	0.12261 (19)	0.0353 (11)	
H7	0.7711	0.6079	0.1279	0.042*	
C8	0.6041 (6)	0.4286 (6)	0.0919 (2)	0.0411 (13)	
H8	0.6653	0.3981	0.0747	0.049*	
C9	0.4511 (6)	0.3490 (6)	0.0865 (2)	0.0374 (13)	
H9	0.4042	0.2593	0.0671	0.045*	
C10	0.3682 (5)	0.4021 (5)	0.10954 (18)	0.0348 (12)	
H10	0.2630	0.3483	0.1051	0.042*	
C11	0.2541 (6)	0.4024 (5)	0.24997 (19)	0.0365 (13)	
H11	0.3111	0.3731	0.2296	0.044*	
C12	0.1780 (6)	0.3163 (5)	0.2920 (2)	0.0383 (13)	
H12	0.1845	0.2307	0.3007	0.046*	
C13	0.0928 (6)	0.3568 (6)	0.3209 (2)	0.0452 (14)	
H13	0.0366	0.2974	0.3491	0.054*	
C14	0.0903 (6)	0.4854 (6)	0.3082 (2)	0.0425 (13)	
H14	0.0332	0.5162	0.3278	0.051*	
C15	0.1714 (5)	0.5678 (5)	0.26678 (17)	0.0282 (10)	
C16	0.1795 (5)	0.7093 (5)	0.25162 (18)	0.0286 (11)	
C17	0.1413 (6)	0.7889 (6)	0.2842 (2)	0.0392 (13)	
H17	0.1033	0.7519	0.3174	0.047*	
C18	0.1594 (7)	0.9260 (6)	0.2675 (2)	0.0486 (15)	
H18	0.1396	0.9862	0.2900	0.058*	
C19	0.2056 (6)	0.9712 (6)	0.2188 (2)	0.0395 (13)	
H19	0.2144	1.0613	0.2062	0.047*	
C20	0.2391 (5)	0.8847 (5)	0.18840 (19)	0.0312 (11)	
H20	0.2716	0.9171	0.1544	0.037*	
C21	−0.0026 (5)	0.3604 (6)	0.1512 (2)	0.0385 (12)	
H21	0.0053	0.3407	0.1862	0.046*	
C22	−0.1253 (6)	0.2570 (6)	0.1241 (2)	0.0435 (14)	
H22	−0.1999	0.1683	0.1400	0.052*	
C23	−0.1365 (6)	0.2856 (6)	0.0737 (2)	0.0456 (14)	
H23	−0.2178	0.2151	0.0536	0.055*	
C24	−0.0301 (6)	0.4160 (6)	0.0526 (2)	0.0405 (13)	
H24	−0.0391	0.4384	0.0180	0.049*	
C25	0.0928 (5)	0.5172 (6)	0.08174 (19)	0.0302 (11)	
C26	0.2129 (5)	0.6598 (5)	0.06185 (18)	0.0305 (11)	
C27	0.2036 (6)	0.7188 (6)	0.01585 (19)	0.0425 (14)	
H27	0.1168	0.6670	−0.0047	0.051*	
C28	0.3202 (7)	0.8532 (6)	−0.0003 (2)	0.0453 (14)	
H28	0.3144	0.8953	−0.0320	0.054*	
C29	0.4451 (6)	0.9257 (6)	0.0299 (2)	0.0438 (14)	
H29	0.5286	1.0173	0.0193	0.053*	
C30	0.4453 (6)	0.8611 (6)	0.07615 (19)	0.0391 (13)	
H30	0.5307	0.9113	0.0974	0.047*	
P1	0.6353 (2)	0.34156 (18)	0.26409 (5)	0.0455 (4)	
F1	0.7904 (4)	0.4131 (6)	0.23794 (18)	0.1132 (18)	
F2	0.6499 (10)	0.2092 (8)	0.2842 (2)	0.210 (4)	
F3	0.7010 (5)	0.4240 (6)	0.31495 (16)	0.1132 (18)	
F4	0.4748 (5)	0.2696 (4)	0.29142 (15)	0.0856 (13)	
F5	0.6100 (5)	0.4687 (5)	0.24542 (17)	0.0983 (15)	
F6	0.5659 (6)	0.2568 (6)	0.21402 (18)	0.147 (2)	
P2	0.03045 (17)	0.98203 (18)	0.07188 (5)	0.0404 (4)	
F7	0.1988 (3)	1.0180 (4)	0.06828 (15)	0.0606 (10)	
F8	−0.0149 (4)	0.8242 (4)	0.09591 (15)	0.0720 (12)	
F9	0.0629 (4)	1.0539 (3)	0.12771 (11)	0.0566 (9)	
F10	−0.1379 (3)	0.9457 (4)	0.07615 (14)	0.0608 (10)	
F11	0.0790 (4)	1.1424 (3)	0.04879 (12)	0.0582 (9)	
F12	−0.0020 (4)	0.9120 (4)	0.01613 (13)	0.0643 (10)	

### Atomic displacement parameters (Å^2^)

**Table:**

	*U*^11^	*U*^22^	*U*^33^	*U*^12^	*U*^13^	*U*^23^
Co1	0.0283 (4)	0.0289 (3)	0.0231 (3)	0.0142 (3)	−0.0001 (3)	0.0007 (3)
N1	0.030 (2)	0.028 (2)	0.026 (2)	0.0130 (19)	−0.0016 (17)	0.0015 (17)
N2	0.031 (2)	0.029 (2)	0.026 (2)	0.0143 (19)	0.0033 (18)	0.0013 (18)
N3	0.032 (2)	0.029 (2)	0.024 (2)	0.0158 (19)	−0.0067 (18)	−0.0026 (17)
N4	0.027 (2)	0.029 (2)	0.027 (2)	0.0162 (18)	0.0002 (17)	0.0004 (18)
N5	0.027 (2)	0.033 (2)	0.026 (2)	0.0156 (19)	−0.0062 (17)	−0.0019 (18)
N6	0.033 (2)	0.034 (2)	0.029 (2)	0.017 (2)	−0.0004 (18)	0.0030 (18)
C1	0.037 (3)	0.033 (3)	0.031 (3)	0.020 (2)	0.003 (2)	0.002 (2)
C2	0.036 (3)	0.031 (3)	0.027 (3)	0.010 (2)	0.003 (2)	−0.003 (2)
C3	0.032 (3)	0.054 (4)	0.036 (3)	0.017 (3)	−0.004 (2)	−0.008 (3)
C4	0.032 (3)	0.049 (3)	0.040 (3)	0.023 (3)	0.003 (2)	−0.007 (3)
C5	0.035 (3)	0.026 (2)	0.021 (2)	0.016 (2)	−0.001 (2)	0.001 (2)
C6	0.031 (3)	0.026 (3)	0.022 (3)	0.012 (2)	0.000 (2)	0.001 (2)
C7	0.036 (3)	0.035 (3)	0.037 (3)	0.020 (3)	0.000 (2)	−0.003 (2)
C8	0.039 (3)	0.041 (3)	0.047 (3)	0.023 (3)	0.010 (3)	−0.003 (3)
C9	0.043 (3)	0.031 (3)	0.038 (3)	0.018 (3)	0.001 (2)	−0.005 (2)
C10	0.027 (3)	0.030 (3)	0.040 (3)	0.008 (2)	−0.002 (2)	−0.003 (2)
C11	0.041 (3)	0.031 (3)	0.040 (3)	0.020 (2)	0.000 (2)	−0.003 (2)
C12	0.047 (3)	0.026 (3)	0.041 (3)	0.018 (3)	−0.002 (3)	0.006 (2)
C13	0.061 (4)	0.042 (3)	0.032 (3)	0.026 (3)	0.007 (3)	0.010 (3)
C14	0.054 (3)	0.050 (3)	0.032 (3)	0.032 (3)	0.012 (3)	0.008 (3)
C15	0.032 (2)	0.031 (3)	0.022 (2)	0.016 (2)	−0.003 (2)	−0.001 (2)
C16	0.022 (2)	0.030 (3)	0.030 (3)	0.010 (2)	0.002 (2)	0.003 (2)
C17	0.059 (4)	0.046 (3)	0.025 (3)	0.036 (3)	0.007 (3)	0.010 (3)
C18	0.072 (4)	0.053 (4)	0.044 (4)	0.049 (3)	−0.001 (3)	−0.004 (3)
C19	0.052 (3)	0.037 (3)	0.040 (3)	0.030 (3)	−0.005 (3)	−0.001 (3)
C20	0.030 (3)	0.032 (3)	0.030 (3)	0.014 (2)	0.001 (2)	0.006 (2)
C21	0.028 (3)	0.044 (3)	0.037 (3)	0.014 (3)	0.002 (2)	0.002 (2)
C22	0.034 (3)	0.041 (3)	0.044 (3)	0.009 (3)	−0.004 (2)	0.004 (3)
C23	0.030 (3)	0.050 (4)	0.045 (4)	0.011 (3)	−0.008 (3)	−0.004 (3)
C24	0.044 (3)	0.052 (4)	0.028 (3)	0.026 (3)	−0.009 (2)	−0.003 (3)
C25	0.027 (3)	0.036 (3)	0.030 (3)	0.018 (2)	0.002 (2)	0.003 (2)
C26	0.033 (3)	0.038 (3)	0.028 (3)	0.023 (2)	0.000 (2)	−0.004 (2)
C27	0.052 (4)	0.053 (4)	0.025 (3)	0.028 (3)	−0.001 (3)	0.005 (3)
C28	0.056 (4)	0.055 (4)	0.028 (3)	0.030 (3)	0.006 (3)	0.010 (3)
C29	0.054 (4)	0.045 (3)	0.032 (3)	0.025 (3)	0.012 (3)	0.008 (2)
C30	0.045 (3)	0.042 (3)	0.024 (3)	0.017 (3)	0.002 (2)	0.002 (2)
P1	0.0655 (11)	0.0525 (10)	0.0310 (9)	0.0388 (9)	0.0047 (7)	0.0015 (7)
F1	0.064 (3)	0.187 (5)	0.088 (3)	0.062 (3)	0.025 (2)	−0.003 (3)
F2	0.424 (12)	0.227 (7)	0.152 (5)	0.293 (9)	0.170 (7)	0.125 (5)
F3	0.094 (3)	0.200 (5)	0.047 (2)	0.075 (4)	−0.028 (2)	−0.044 (3)
F4	0.089 (3)	0.077 (3)	0.074 (3)	0.029 (2)	0.034 (2)	0.009 (2)
F5	0.126 (4)	0.107 (4)	0.101 (3)	0.087 (3)	0.032 (3)	0.055 (3)
F6	0.148 (5)	0.124 (4)	0.074 (3)	−0.004 (4)	0.016 (3)	−0.057 (3)
P2	0.0351 (8)	0.0404 (8)	0.0340 (8)	0.0101 (6)	−0.0021 (6)	−0.0015 (7)
F7	0.0390 (19)	0.050 (2)	0.081 (3)	0.0129 (16)	−0.0036 (18)	−0.0054 (18)
F8	0.073 (3)	0.0357 (19)	0.087 (3)	0.0123 (18)	0.017 (2)	0.0097 (18)
F9	0.067 (2)	0.056 (2)	0.0326 (17)	0.0197 (18)	−0.0080 (15)	−0.0012 (14)
F10	0.0332 (17)	0.068 (2)	0.066 (2)	0.0142 (17)	−0.0034 (16)	−0.0194 (19)
F11	0.072 (2)	0.0511 (19)	0.0422 (19)	0.0244 (19)	−0.0013 (18)	0.0096 (16)
F12	0.060 (2)	0.072 (2)	0.048 (2)	0.0230 (19)	−0.0049 (17)	−0.0207 (19)

### Geometric parameters (Å, º)

**Table:**

Co1—N2	2.117 (4)	C14—C15	1.374 (7)
Co1—N3	2.123 (4)	C14—H14	0.9500
Co1—N6	2.124 (4)	C15—C16	1.479 (6)
Co1—N4	2.124 (4)	C16—C17	1.374 (7)
Co1—N5	2.139 (4)	C17—C18	1.405 (7)
Co1—N1	2.146 (4)	C17—H17	0.9500
N1—C5	1.342 (6)	C18—C19	1.357 (7)
N1—C1	1.345 (6)	C18—H18	0.9500
N2—C6	1.340 (6)	C19—C20	1.366 (7)
N2—C10	1.349 (6)	C19—H19	0.9500
N3—C11	1.336 (6)	C20—H20	0.9500
N3—C15	1.347 (6)	C21—C22	1.379 (7)
N4—C20	1.335 (6)	C21—H21	0.9500
N4—C16	1.342 (6)	C22—C23	1.367 (7)
N5—C21	1.324 (6)	C22—H22	0.9500
N5—C25	1.352 (6)	C23—C24	1.362 (7)
N6—C30	1.324 (6)	C23—H23	0.9500
N6—C26	1.344 (6)	C24—C25	1.402 (7)
C1—C2	1.368 (6)	C24—H24	0.9500
C1—H1	0.9500	C25—C26	1.469 (7)
C2—C3	1.385 (7)	C26—C27	1.374 (6)
C2—H2	0.9500	C27—C28	1.375 (7)
C3—C4	1.382 (7)	C27—H27	0.9500
C3—H3	0.9500	C28—C29	1.374 (8)
C4—C5	1.359 (6)	C28—H28	0.9500
C4—H4	0.9500	C29—C30	1.383 (7)
C5—C6	1.493 (6)	C29—H29	0.9500
C6—C7	1.374 (6)	C30—H30	0.9500
C7—C8	1.378 (7)	P1—F6	1.539 (5)
C7—H7	0.9500	P1—F3	1.542 (4)
C8—C9	1.380 (7)	P1—F2	1.544 (5)
C8—H8	0.9500	P1—F5	1.545 (4)
C9—C10	1.368 (7)	P1—F1	1.551 (4)
C9—H9	0.9500	P1—F4	1.609 (4)
C10—H10	0.9500	P2—F12	1.587 (4)
C11—C12	1.386 (7)	P2—F8	1.587 (4)
C11—H11	0.9500	P2—F10	1.592 (3)
C12—C13	1.376 (7)	P2—F7	1.592 (4)
C12—H12	0.9500	P2—F11	1.594 (4)
C13—C14	1.385 (7)	P2—F9	1.596 (3)
C13—H13	0.9500		
			
N2—Co1—N3	96.86 (15)	C14—C15—C16	122.7 (4)
N2—Co1—N6	90.63 (15)	N4—C16—C17	121.6 (4)
N3—Co1—N6	168.13 (15)	N4—C16—C15	115.9 (4)
N2—Co1—N4	169.58 (15)	C17—C16—C15	122.5 (4)
N3—Co1—N4	77.66 (14)	C16—C17—C18	118.7 (5)
N6—Co1—N4	96.28 (15)	C16—C17—H17	120.6
N2—Co1—N5	96.15 (15)	C18—C17—H17	120.6
N3—Co1—N5	92.62 (14)	C19—C18—C17	119.0 (5)
N6—Co1—N5	77.38 (15)	C19—C18—H18	120.5
N4—Co1—N5	92.97 (14)	C17—C18—H18	120.5
N2—Co1—N1	77.02 (15)	C18—C19—C20	118.8 (5)
N3—Co1—N1	94.01 (14)	C18—C19—H19	120.6
N6—Co1—N1	96.65 (15)	C20—C19—H19	120.6
N4—Co1—N1	94.39 (15)	N4—C20—C19	123.3 (5)
N5—Co1—N1	171.00 (15)	N4—C20—H20	118.3
C5—N1—C1	118.6 (4)	C19—C20—H20	118.3
C5—N1—Co1	115.2 (3)	N5—C21—C22	123.6 (5)
C1—N1—Co1	125.9 (3)	N5—C21—H21	118.2
C6—N2—C10	117.8 (4)	C22—C21—H21	118.2
C6—N2—Co1	116.1 (3)	C23—C22—C21	118.1 (5)
C10—N2—Co1	125.6 (3)	C23—C22—H22	120.9
C11—N3—C15	118.6 (4)	C21—C22—H22	120.9
C11—N3—Co1	125.6 (3)	C24—C23—C22	119.5 (5)
C15—N3—Co1	114.3 (3)	C24—C23—H23	120.3
C20—N4—C16	118.4 (4)	C22—C23—H23	120.3
C20—N4—Co1	126.2 (3)	C23—C24—C25	120.1 (5)
C16—N4—Co1	114.6 (3)	C23—C24—H24	119.9
C21—N5—C25	118.8 (4)	C25—C24—H24	119.9
C21—N5—Co1	126.6 (3)	N5—C25—C24	119.8 (4)
C25—N5—Co1	114.1 (3)	N5—C25—C26	116.5 (4)
C30—N6—C26	118.5 (4)	C24—C25—C26	123.7 (5)
C30—N6—Co1	125.9 (3)	N6—C26—C27	121.2 (5)
C26—N6—Co1	115.5 (3)	N6—C26—C25	115.8 (4)
N1—C1—C2	123.0 (5)	C27—C26—C25	123.0 (5)
N1—C1—H1	118.5	C26—C27—C28	119.9 (5)
C2—C1—H1	118.5	C26—C27—H27	120.1
C1—C2—C3	117.9 (5)	C28—C27—H27	120.1
C1—C2—H2	121.1	C29—C28—C27	119.1 (5)
C3—C2—H2	121.1	C29—C28—H28	120.5
C4—C3—C2	119.1 (5)	C27—C28—H28	120.5
C4—C3—H3	120.5	C28—C29—C30	117.8 (5)
C2—C3—H3	120.5	C28—C29—H29	121.1
C5—C4—C3	120.0 (5)	C30—C29—H29	121.1
C5—C4—H4	120.0	N6—C30—C29	123.4 (5)
C3—C4—H4	120.0	N6—C30—H30	118.3
N1—C5—C4	121.4 (4)	C29—C30—H30	118.3
N1—C5—C6	115.3 (4)	F6—P1—F3	178.5 (3)
C4—C5—C6	123.3 (4)	F6—P1—F2	90.5 (4)
N2—C6—C7	121.7 (4)	F3—P1—F2	89.2 (4)
N2—C6—C5	115.6 (4)	F6—P1—F5	89.3 (3)
C7—C6—C5	122.6 (4)	F3—P1—F5	90.9 (3)
C6—C7—C8	119.9 (5)	F2—P1—F5	176.2 (4)
C6—C7—H7	120.0	F6—P1—F1	88.5 (3)
C8—C7—H7	120.0	F3—P1—F1	92.9 (3)
C7—C8—C9	118.6 (5)	F2—P1—F1	92.1 (4)
C7—C8—H8	120.7	F5—P1—F1	91.7 (3)
C9—C8—H8	120.7	F6—P1—F4	91.9 (3)
C10—C9—C8	118.6 (5)	F3—P1—F4	86.6 (2)
C10—C9—H9	120.7	F2—P1—F4	88.6 (3)
C8—C9—H9	120.7	F5—P1—F4	87.6 (2)
N2—C10—C9	123.2 (5)	F1—P1—F4	179.2 (3)
N2—C10—H10	118.4	F12—P2—F8	90.4 (2)
C9—C10—H10	118.4	F12—P2—F10	90.0 (2)
N3—C11—C12	122.2 (5)	F8—P2—F10	90.2 (2)
N3—C11—H11	118.9	F12—P2—F7	90.5 (2)
C12—C11—H11	118.9	F8—P2—F7	89.4 (2)
C13—C12—C11	119.0 (5)	F10—P2—F7	179.4 (2)
C13—C12—H12	120.5	F12—P2—F11	90.7 (2)
C11—C12—H12	120.5	F8—P2—F11	178.6 (2)
C12—C13—C14	118.9 (5)	F10—P2—F11	90.6 (2)
C12—C13—H13	120.5	F7—P2—F11	89.8 (2)
C14—C13—H13	120.5	F12—P2—F9	179.4 (2)
C15—C14—C13	119.1 (5)	F8—P2—F9	90.2 (2)
C15—C14—H14	120.5	F10—P2—F9	89.79 (19)
C13—C14—H14	120.5	F7—P2—F9	89.68 (19)
N3—C15—C14	122.1 (4)	F11—P2—F9	88.70 (19)
N3—C15—C16	115.2 (4)		
			
N2—Co1—N1—C5	−3.2 (3)	C10—N2—C6—C7	3.6 (7)
N3—Co1—N1—C5	92.9 (3)	Co1—N2—C6—C7	−169.0 (4)
N6—Co1—N1—C5	−92.3 (3)	C10—N2—C6—C5	−179.1 (4)
N4—Co1—N1—C5	170.8 (3)	Co1—N2—C6—C5	8.2 (5)
N2—Co1—N1—C1	−176.9 (4)	N1—C5—C6—N2	−11.1 (6)
N3—Co1—N1—C1	−80.8 (4)	C4—C5—C6—N2	168.7 (5)
N6—Co1—N1—C1	94.0 (4)	N1—C5—C6—C7	166.1 (4)
N4—Co1—N1—C1	−2.9 (4)	C4—C5—C6—C7	−14.1 (7)
N3—Co1—N2—C6	−95.6 (3)	N2—C6—C7—C8	−1.4 (7)
N6—Co1—N2—C6	93.7 (3)	C5—C6—C7—C8	−178.4 (4)
N4—Co1—N2—C6	−38.0 (10)	C6—C7—C8—C9	−2.3 (7)
N5—Co1—N2—C6	171.0 (3)	C7—C8—C9—C10	3.6 (8)
N1—Co1—N2—C6	−3.0 (3)	C6—N2—C10—C9	−2.2 (7)
N3—Co1—N2—C10	92.5 (4)	Co1—N2—C10—C9	169.6 (4)
N6—Co1—N2—C10	−78.3 (4)	C8—C9—C10—N2	−1.4 (8)
N4—Co1—N2—C10	150.0 (7)	C15—N3—C11—C12	1.3 (7)
N5—Co1—N2—C10	−0.9 (4)	Co1—N3—C11—C12	−164.1 (4)
N1—Co1—N2—C10	−175.0 (4)	N3—C11—C12—C13	1.4 (8)
N2—Co1—N3—C11	−14.2 (4)	C11—C12—C13—C14	−2.4 (8)
N6—Co1—N3—C11	114.6 (8)	C12—C13—C14—C15	0.8 (8)
N4—Co1—N3—C11	174.8 (4)	C11—N3—C15—C14	−3.0 (7)
N5—Co1—N3—C11	82.4 (4)	Co1—N3—C15—C14	164.0 (4)
N1—Co1—N3—C11	−91.5 (4)	C11—N3—C15—C16	176.7 (4)
N2—Co1—N3—C15	179.9 (3)	Co1—N3—C15—C16	−16.3 (5)
N6—Co1—N3—C15	−51.3 (9)	C13—C14—C15—N3	2.0 (8)
N4—Co1—N3—C15	8.9 (3)	C13—C14—C15—C16	−177.7 (5)
N5—Co1—N3—C15	−83.6 (3)	C20—N4—C16—C17	−0.3 (7)
N1—Co1—N3—C15	102.5 (3)	Co1—N4—C16—C17	170.2 (4)
N2—Co1—N4—C20	111.0 (8)	C20—N4—C16—C15	−179.5 (4)
N3—Co1—N4—C20	170.1 (4)	Co1—N4—C16—C15	−9.1 (5)
N6—Co1—N4—C20	−20.3 (4)	N3—C15—C16—N4	17.1 (6)
N5—Co1—N4—C20	−97.9 (4)	C14—C15—C16—N4	−163.2 (5)
N1—Co1—N4—C20	76.9 (4)	N3—C15—C16—C17	−162.1 (4)
N2—Co1—N4—C16	−58.6 (9)	C14—C15—C16—C17	17.6 (7)
N3—Co1—N4—C16	0.5 (3)	N4—C16—C17—C18	−2.3 (8)
N6—Co1—N4—C16	170.2 (3)	C15—C16—C17—C18	176.9 (5)
N5—Co1—N4—C16	92.5 (3)	C16—C17—C18—C19	3.9 (9)
N1—Co1—N4—C16	−92.7 (3)	C17—C18—C19—C20	−2.8 (9)
N2—Co1—N5—C21	87.5 (4)	C16—N4—C20—C19	1.4 (7)
N3—Co1—N5—C21	−9.7 (4)	Co1—N4—C20—C19	−167.8 (4)
N6—Co1—N5—C21	176.8 (4)	C18—C19—C20—N4	0.2 (8)
N4—Co1—N5—C21	−87.5 (4)	C25—N5—C21—C22	1.9 (7)
N2—Co1—N5—C25	−84.1 (3)	Co1—N5—C21—C22	−169.4 (4)
N3—Co1—N5—C25	178.7 (3)	N5—C21—C22—C23	0.0 (8)
N6—Co1—N5—C25	5.2 (3)	C21—C22—C23—C24	−2.1 (8)
N4—Co1—N5—C25	100.9 (3)	C22—C23—C24—C25	2.4 (8)
N2—Co1—N6—C30	−88.2 (4)	C21—N5—C25—C24	−1.5 (7)
N3—Co1—N6—C30	142.5 (7)	Co1—N5—C25—C24	170.8 (4)
N4—Co1—N6—C30	84.0 (4)	C21—N5—C25—C26	178.3 (4)
N5—Co1—N6—C30	175.7 (4)	Co1—N5—C25—C26	−9.4 (5)
N1—Co1—N6—C30	−11.1 (4)	C23—C24—C25—N5	−0.6 (7)
N2—Co1—N6—C26	96.2 (3)	C23—C24—C25—C26	179.6 (5)
N3—Co1—N6—C26	−33.1 (9)	C30—N6—C26—C27	−2.9 (7)
N4—Co1—N6—C26	−91.6 (3)	Co1—N6—C26—C27	173.1 (4)
N5—Co1—N6—C26	0.1 (3)	C30—N6—C26—C25	179.1 (4)
N1—Co1—N6—C26	173.2 (3)	Co1—N6—C26—C25	−4.9 (5)
C5—N1—C1—C2	−2.1 (7)	N5—C25—C26—N6	9.7 (6)
Co1—N1—C1—C2	171.4 (4)	C24—C25—C26—N6	−170.5 (5)
N1—C1—C2—C3	0.2 (7)	N5—C25—C26—C27	−168.3 (5)
C1—C2—C3—C4	1.1 (8)	C24—C25—C26—C27	11.5 (7)
C2—C3—C4—C5	−0.4 (8)	N6—C26—C27—C28	1.9 (8)
C1—N1—C5—C4	2.8 (7)	C25—C26—C27—C28	179.8 (5)
Co1—N1—C5—C4	−171.4 (4)	C26—C27—C28—C29	0.5 (8)
C1—N1—C5—C6	−177.5 (4)	C27—C28—C29—C30	−1.8 (8)
Co1—N1—C5—C6	8.3 (5)	C26—N6—C30—C29	1.5 (7)
C3—C4—C5—N1	−1.5 (8)	Co1—N6—C30—C29	−174.0 (4)
C3—C4—C5—C6	178.7 (4)	C28—C29—C30—N6	0.8 (8)

### Hydrogen-bond geometry (Å, º)

**Table:**

*D*—H···*A*	*D*—H	H···*A*	*D*···*A*	*D*—H···*A*
C24—H24···F2^i^	0.95	2.49	3.324 (7)	146
C23—H23···F4^i^	0.95	2.55	3.253 (7)	131
C18—H18···F11^ii^	0.95	2.52	3.149 (6)	124
C13—H13···F10^iii^	0.95	2.50	3.208 (6)	131
C10—H10···F11^iv^	0.95	2.51	3.265 (6)	137
C9—H9···F7^iv^	0.95	2.33	3.136 (6)	142
C7—H7···F8^v^	0.95	2.38	3.160 (7)	139
C2—H2···F2^vi^	0.95	2.33	3.081 (7)	136

Symmetry codes: (i) −*y*, *x*−*y*, *z*−1/3; (ii) −*x*+*y*−1, −*x*+1, *z*+1/3; (iii) −*x*+*y*−1, −*x*, *z*+1/3; (iv) *x*, *y*−1, *z*; (v) *x*+1, *y*, *z*; (vi) *x*, *y*+1, *z*.
